# Cross reactive cellular immune responses in chickens previously exposed to low pathogenic avian influenza

**DOI:** 10.1186/1753-6561-5-S4-S13

**Published:** 2011-06-03

**Authors:** Darrell R Kapczynski, Karen Liljebjelke, Gururaj Kulkarni, Henry Hunt, Hai Jun Jiang, Daniel Petkov

**Affiliations:** 1 Southeast Poultry Research Laboratory, Agricultural Research Service, USA, Department of Agriculture, 934 College Station Road, Athens, GA 30605, USA; 2 Avian Disease and Oncology Laboratory, Agricultural Research Service, USA, Department of Agriculture, 3606 East Mount Hope Road, East Lansing, Michigan, 48823, USA

## Abstract

**Background:**

Avian influenza (AI) infection in poultry can result in high morbidity and mortality, and negatively affect international trade. Because most AI vaccines used for poultry are inactivated, our knowledge of immunity against AI is based largely on humoral immune responses. In fact, little is known about cellular immunity following a primary AI infection in poultry, especially regarding cytotoxic T lymphocytes (CTL’s).

**Methods:**

In these studies, major histocompatibility complex (MHC)-defined (B^2^/B^2^) chickens were infected with low pathogenic AI (LPAI) H9N2 and clinical signs of disease were monitored over a two weeks period. Splenic lymphocytes from infected and naïve birds were examined for cross reactivity against homologous and heterologous (H7N2) LPAI by ex vivo stimulation. Cellular immunity was determined by cytotoxic lysis of B^2^/B^2^ infected lung target cells and proliferation of T cells following exposure to LPAI.

**Results:**

Infection with H9N2 resulted in statistically significant weight loss compared to sham-infected birds. Splenic lymphocytes derived from H9N2-infected birds displayed lysis of both homologous (H9N2) and heterologous (H7N2) infected target cells, whereas lymphocytes obtained from sham-infected birds did not. T cell proliferation was determined to be highest when exposed to the homologous virus.

**Conclusions:**

Taken together these data extend the findings that cellular immunity, including CTL’s, is cross reactive against heterologous isolates of AI and contribute to protection following infection.

## Background

Cell-mediated immunity (CMI) is antigen specific immunity mediated by T lymphocytes and has been suggested to be an important factor to the development of protection in chickens vaccinated against viral diseases [1]. The subsets of T lymphocytes: CD4+ helper cells and CD8+ cytotoxic cells constitute the principal cells of the CMI response. For influenza, CD8+ CTL’s play a crucial role in controlling infectious virus from the lungs of mice [2][3]. A number of recent studies have provided evidence that CMI directed against viral epitopes conserved among influenza A viruses, such as those within the nucleoprotein (NP) and hemagglutinin (HA), may contribute to protection against influenza [4][5]. In fact, influenza virus NP-specific CTL’s generated through vaccination or introduced by adoptive transfer enhance viral clearance and lead to recovery of the host and protection from death [6]. The objectives of this study were to determine the level of cross reactive CMI against homologous and heterologous isolates of LPAI.

## Methods

### Birds

Two-day-of age chickens were received from the USDA-ARS-Avian Disease Oncology Laboratory (East Lansing, Michigan) containing the B^2^/B^2^ defined MHC allele (Line 15.6-2). Birds were placed in Horsfall units in BSL3E facilities (USDA-ARS, Athens, Georgia, USA) and provided feed and water *ad libitum*.

### Viruses

Low pathogenic AI viruses of H9N2 (A/Chicken/NJ/12220/97) and H7N2 (A/Turkey/Virginia/4259/02) subtypes were propagated in the allantoic cavities of 9-11 day of embryonation SPF chicken eggs. Viral titers were determined as previously described [7].

### H9N2 infection

Two-week-old chickens (6 per group) were inoculated via the intranasal route with 10^7^ EID_50_ of H9N2 AI virus or sham inoculum in 0.2 ml volume (1/2 each nare) at 2 weeks of age. Chicken were monitored daily for clinical signs of disease and weight loss monitored at 2, 4 and 7 days post inoculation (p.i.) following H9N2 primary infection. Mean weight was compared by ANOVA using the Tukey Test (SigmaStat).

### CTL lysis of target cells

Splenic lymphocytes were harvested from B^2^/B^2^ infected and control chickens, three each, at 10 days post- infection as previously described [8]. MHC-matched lung cells were obtained as previously described and used as target cells for CTL assay [9]. Lung cells were infected with either H9N2 or H7N2 AI at an MOI of 2 for 16 hours prior to testing. CTL activity was monitored using the CytoTox96 nonradioactive assay (Promega) with the % cells lysed determined by detection of cytosolic lactate dehydrogenase in supernatants from control and infected target cells.

### Proliferation

Lymphocyte proliferation as a measure of cellular memory was performed with almarBlue™ (Invitrogen) as previously described, using BPL-inactivated H9N2 (5 µg/ml) or H7N2 (5 µg/ml) [8]. The T cell mitogen, concanavalin A (5 µg/ml), was used as a positive control.

## Results

### Weight loss following H9N2 infection

**Figure 1:**
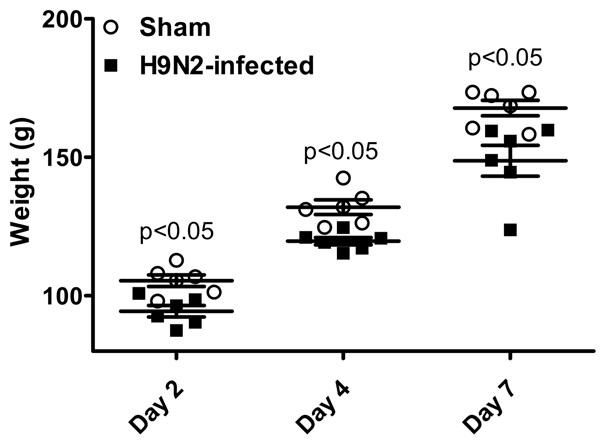
**Effect of A/chicken/NJ/97 (H9N2) infection on weight gain in MHC-defined (B^2^/B^2^) chickens at 2, 4 and 7 days post infection**. Two-week old birds received either PBS (Sham) or 10^7^ EID_50_ H9N2 per bird via intranasal route. Bird weight was significantly reduced between the two groups on each day tested (p<0.05).

Infection with A\Chicken\NJ\97 (H9N2) following a natural route of exposure (intranasal) significantly decreased weight gains at day 2, 4 and 7 post-inoculation compared to control (uninfected) chickens (Fig 1). In general, H9N2 infected birds weighed approximately 10% less than the sham inoculated group at each time point. Apart from weight loss, overt signs of clinical disease or respiratory distress were not observed in the H9N2 challenged group throughout the course of the study.

### Lysis of homologous and heterologous infected B^2^/B^2^ lung cells

**Figure 2:**
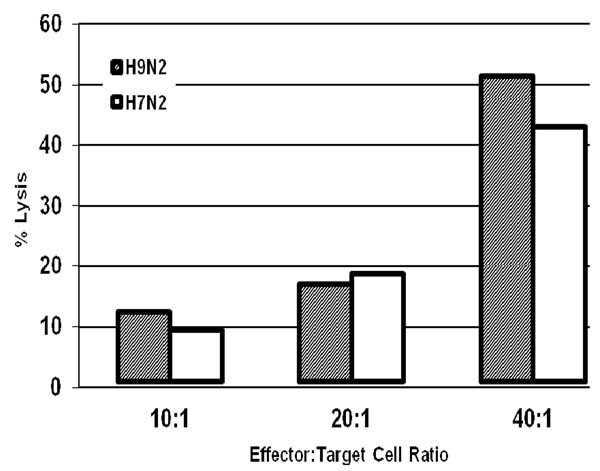
**Recognition of homologous and heterologous LPAI ex vivo by splenocytes derived from H9N2-infected chickens**. The cross-reactivity of lymphocytes was tested with MHC- matched B2/B2 lung cell cultures infected with H9N2 or H7N2 LPAI. Effector:target (E:T) ratios were 10, 20 and 40 for all subjects. Standard deviation of the means was <10%. Spontaneous lysis from splenocytes derived from naive birds were <7 % against either virus (data not shown).

Splenic T cells from inbred chickens infected with H9N2 AI lysed target cells infected with either homologous or H7N2 AI at all effector:target (E:T) ratios tested (Fig 2). A dose response based on E:T ratios was observed. At E:T ratio of 40:1, 52 % of H9N2-infected B^2^/B^2^ lung target cells were lysed, while 43 % of H7N2-infected target cells were lysed. At lower E:T ratios, approximately 18 % (20:1) and 10 % (10:1) of infected target cells were lysed in either H9N2 or H7N2-infected lung cells. The splenic T cells from H9N2 infected birds did not lyse uninfected target cells, although spontaneous lysis was approximately 7 % (data not shown). In addition, lymphocytes obtained from sham-infected birds did not lyse infected or control lung cell cultures (data not shown).

### Proliferation

**Figure 3:**
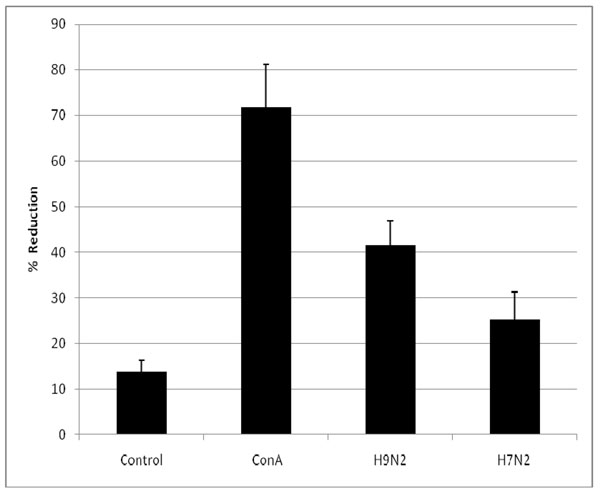
**Lymphocyte proliferation responses induced by H9N2 infection in MHC-defined chickens against homologous and heterologous AI.** Groups of chickens received either sham or H9N2 infection. Spleens of individual birds were removed, and lymphocyte proliferation was evaluated using the alamarBlue™ method. Samples were read at 48 hours and the percent alamarBlue™ reduced, as an indicator of proliferation, presented per group. Proliferation by lymphocytes from sham birds against either AI virus was <12 % (data not shown).

Splenocytes from H9N2 infected birds were tested for CMI memory response against homologous and heterologous AI via lymphocyte proliferative. The results (Fig. 3) indicate an increased proliferation response, as determined as an increase in the percent of alamaBlue™ reduced by the lymphocytes, against both the H9N2 and H7N2 virus. The highest proliferative response was against the homologous virus (42%), which was less than the response to the T-cell mitogen, concanavalin A (ConA). The T cell proliferative response to the heterologous virus was approximately half (25%) of that observed against the homologous virus.

## Discussion

The main objective of the present research was to examine CMI following infection with low pathogenic AI, and to identify cross reactive T cell immunity in chickens. Using birds with a genetically defined MHC haplotype (B^2^/B^2^), we found CTL’s isolated from H9N2 infected birds were active against homologous and heterologous isolates of AI based on lysis of MHC-matched target cells and T cell proliferation. In addition, memory T cells demonstrated proliferation in the presence of either isolate tested. These data suggest that antigenic peptides found in both isolates are presented by MHC-class I and II molecules that lead to production of memory CTL’s and stimulated proliferation of T cells, respectively. The proteins containing these peptides remain to be determined, although at least one report has identified a T cell epitope on the HA protein which could activate both chicken CD4^+^ and CD8^+^ cells [4].

For this study birds infected with a LPAI H9N2 isolate were the source of lymphocytes and examining cross reactivity against a heterologous H7N2 isolate. We provide evidence that the H9N2 AI virus can induce cross reactive CMI against the H7N2 AI isolate. Because the majority of vaccines used across the world to protect poultry from AI are inactivated, they must be matched to the field subtype. While these vaccines induce protective antibodies against homologous subtypes based on HA, little to no protective cross reactivity is afforded against heterologous isolates [10]. In contrast, Seo et al. was able to adaptively transfer CD8^+^ lymphocytes from H9N2-infected chickens and demonstrate protection from H5N1 lethal challenge [9]. Whether the cross reactive CMI demonstrated here could protect against a H7N2 challenge in vivo, remains to be determined.

## Conclusions

Taken together these data extend the findings that CMI, including production of CTL’s, is cross reactive against heterologous isolates of AI infection, and contribute to protection following infection.

## Competing interests

The authors declare that they have no competing interests.

## Authors' contributions

DRK designed the experiments, carried out the analysis, and drafted the manuscript. KL and DP performed the animal experiments in BSL3E, and together with HJ helped perform the ex vivo experiments. HH and GK developed the model and provided expertise with the MHC birds. All authors approved the final manuscript.
